# A nationwide study of asthma correlates among adolescents in Saudi Arabia

**DOI:** 10.1186/s40733-020-00056-8

**Published:** 2020-06-01

**Authors:** Umayya Musharrafieh, Hani Tamim, Rana Houry, Fadia AlBuhairan

**Affiliations:** 1 Department of Family Medicine, American University of Beirut Medical Center, Beirut, Lebanon; 2 Department of Internal Medicine, American University of Beirut Medical Center, Beirut, Lebanon; 3 Department of Pediatrics and Adolescent Medicine, AlDara Hospital and Medical Center; College of Medicine, Alfaisal University, Riyadh, Saudi Arabia

**Keywords:** Asthma, Comorbidities, Saudi Arabia, Lifestyle, Adolescents

## Abstract

**Background:**

Asthma is a chronic airway inflammation disease that is frequently found in children and adolescents with an increasing prevalence. Several studies are linking its presence to many lifestyle and health correlates. The objective of this study was to explore these correlates and find characteristics of self-reported asthmatics among adolescents in Saudi Arabia.

**Methods:**

This is a cross-sectional, school-based study carried out in all 13 regions of Saudi Arabia. Sampling was randomly done from intermediate and secondary school students. Data in our study consisted of demographic characteristics, health conditions and lifestyle patterns and were compared between the two groups: asthmatics versus non-asthmatics. Comparison between the two groups was done by analyzing our data using Statistical Analysis Software SURVEYFREQ procedure (SAS Version 9; SAS Institute, Cary, NC).

**Results:**

Among a sample of 11,348 participants, the prevalence of self-reported asthma was found to be 8.2%. Various characteristics were found significantly different between the 2 groups including the gender, the weight, the family’s education, and dietary patterns. Self –reported asthmatic were more likely to be males, overweight or obese, with a lower father’s level of education and a higher consumption of milk and power drinks.

**Conclusion:**

Asthma disease remains prevalent among adolescents in Saudi Arabia and requires higher awareness and better guidance for its prevention and treatment. Further efforts should focus on health promotion and lifestyle wellness to support preventive efforts of this chronic disease condition.

## Background

Asthma is a chronic inflammatory disease that affects the airways of the lungs. It is characterized by a hyper-responsiveness of the intrapulmonary airways and a variable resistance to airflow that could be reversible spontaneously or in response to treatment [1].

The World Health Organization estimates that 235 million people worldwide of all ages suffer from asthma [2]. With the modernization of the lifestyles and consequent urbanization,this prevalence remains on an increase [3]. According to the National Institute of Health, the prevalence and severity of asthma is well associated with gender differences, with asthma being more common among males during childhood [4]. Besides its increasing prevalence, asthma has been linked to several health and lifestyle correlates, including the increasing technology and social media use by adolescents; asthmatic children have been found to use technology and spend more time on social media than non-asthmatics [5].

Asthma is among the top twenty chronic conditions for global ranking of disability-adjusted life years in children; in the mid-childhood ages 5–14 years it is among the top 10 causes. The burden of asthma on patients, family, and society is inexplicably high in low-income and middle income countries, where access to adequate treatment is not available. Although there is a worldwide downward trend of asthma mortality in adults and children over the past 25 years, a wide global disparity remains in years of life lost because of asthma [6].

In a recent meta-analysis, many higher comorbid conditions were found to be associated with asthma, such as cardiovascular disease [7, 8], hypertension [9], diabetes [10], allergies [11], obesity [12], metabolic and endocrine conditions [13], gastro-esophageal reflux disease [14], and urinary tract conditions [13]. Not sparing psychological aspects, adolescents with asthma are more likely to have depression and reduced quality of life [15], and are at increased risk of suffering from behavioral and psychological problems including school and cyber bullying [5].

Modern lifestyle factors and diet habits have also impacted asthma disease and frequency. While the wide fast food consumption, frequent soda intake, sedentary lifestyle and lack of physical activity and exercise have been associated with an increased asthma prevalence; milk, fruits and fish intake may have a protective association with asthma [16–18].

At another level, school performance is well associated and affected by asthma. Several studies have revealed presence of significant school problems in asthmatic children [19], and this has been partly attributed to interrupted sleep due to nocturnal asthma and nighttime awakening in affected children [20].

Although the prevalence of asthma in Saudi Arabian children and adolescents has been reported to range from 8 to 25% based on studies conducted over the past three decades [21], the characteristics of children and adolescents with asthma in relation to disease and lifestyle patterns have not been explored. Most surveys have addressed one aspect or characteristic in relation to asthma status at a time.

The objective of this study was to assess, among adolescents in Saudi Arabia, factors associated with self-reported asthma, including demographic characteristics, diet, lifestyle factors, psychological state and comorbid diseases.

## Methods

### Jeeluna® study

A school-based, cross-sectional study was conducted in all 13 regions of the Kingdom of Saudi Arabia (KSA) in 2011–2012 aiming to identify health risk behaviors and status among adolescents in KSA. The original sample size was calculated to be 12,000 students in intermediate and secondary schools. With a prevalence of 30%, a 1% margin of error and a 99% confidence, a sample size to estimate proportions of population characteristics was determined to be 11,361. It was further increased by 5% to justify contingencies such as no response or missing answers. Results and details of the original study were published elsewhere [22]. However, in brief, the original study assessed adolescents’ health risk behaviors and health status, clinical anthropometric measurements, and laboratory investigations using data from self-administrated questionnaires in addition to data collected by trained data-collecting teams. Schools were randomly selected from a Ministry of Education (MOE) list of registered schools, which included gender segregated intermediate and secondary schools. Sample was stratified by gender, level (intermediate or secondary), school district, and region. Various health risk behaviors were addressed, including dietary behaviors, tobacco use, bullying, sedentary lifestyle lack of safety measures, as well as the presence of comorbidities. The study was reviewed and approved by the ethics committees at King Abdullah International Medical Research Center and the MOE. Also permission was obtained from schools, and consents and assents from parents and students were obtained, respectively.

Study design:

The design of our project was a nested study for a secondary data analysis using data from the above mentioned cross-sectional survey.

For the current study, our inclusion criteria were: (1) participants aged between 10 and 18 years and (2) available information on whether individual participant was asthmatic or not. This gave us a sample of 11,348 participants. Presence of asthma was based on adolescents’ self-report of whether or not they had a diagnosis of asthma.

### Data collection

For our study, we extracted relevant data from the original database used in the original study. Our final data mainly consisted of: (1) demographic characteristics such as age, gender and family and economical status; (2) health conditions including comorbidities and BMIs, (3) dietary behaviors such as number of meals and different food intake, (4) violence and bullying, (5) sleep patterns, (6) tobacco use, (7) education and school performance, and (8) activities including physical activity and technology use. Body Mass Index (BMI) was categorized as it is in the Center for Disease Control and Prevention BMI charts [23] considering percentiles <5th as underweight, 5th to <85th as healthy weight, 85th to <95th as overweight and ≥ 95th as obese. As for daily dietary patterns, 2–4 meals per day and 1–4 snacks per day were considered as a regular diet [24, 25].

### Statistical analysis

Participants were divided in 2 groups based on presence or absence of asthma. Data were analyzed and adjusted for complex sampling design using Statistical Analysis Software SURVEYFREQ procedure (SAS Version 9; SAS Institute, Cary, NC). For categorical variables, we calculated frequencies and percentages, as for continuous variables, we considered means and standard deviations. Asthmatic and non-asthmatic patients’ differences were detected using the chi-square test. Multivariate logistic regression analyses were carried out to identify predictors of asthma. Results were reported as odds ratio (OR), and 95% confidence interval (CI). A *p*-value < 0.05 was used to indicate statistical significance.

## Results

### Participant’s characteristics and comorbidities

Our final sample included 11,348 participants, with a prevalence of self-reported asthma reaching 8.2% (95% CI: 7.7–8.8%). Fifty one percent of the participants were males, and 28.4% were aged between 10 and 14 years. Participants’ demographic characteristics and their comorbidities are presented in Table 1.

**Table 1:** Demographic characteristics and comorbidities of asthmatics and healthy controls

		All	Non-asthmatic	Asthmatic	
		(*n* = 11,348) %	(*n* = 10,412) %	(*n* = 936) %	*p*-value
**Demographics**					
Age (years)	Between 10 and 14	3209 (28.4)	2930 (28.2)	279 (29.9)	0.28
	Between 15 and 18	8101 (71.6)	7447 (71.8)	654 (70.1)	
Gender	Male	5808 (51.2)	5197 (49.9)	611 (65.3)	< 0.001
	Female	5540 (48.8)	5215 (50.1)	325 (34.7)	
Nationality	Saudi	9632 (87.3)	8794 (87.0)	838 (90.7)	0.001
	Non Saudi	1400 (12.7)	1314 (13.0)	86 (9.3)	
Father’s level of education: high school and above		5785 (59.4)	5343 (59.8)	442 (54.6)	0.003
Mother’s level of education: high school and above		4563 (45.1)	4213 (45.3)	350 (42.7)	0.16
Monthly income for family ≤15,000 SAR		4165 (71.0)	3768 (70.7)	397 (74.8)	0.05
**Comorbidities**					
Diabetes		76 (0.7)	70 (0.7)	6 (0.7)	0.95
Hematological disorder		419 (3.7)	374 (3.6)	45 (4.9)	0.05
Allergies other than asthma		531 (4.7)	418 (4.0)	113 (12.2)	< 0.001
Genitourinary		137 (1.2)	116 (1.1)	21 (2.3)	0.002
BMI	Underweight	1704 (15.1)	1559 (15.1)	145 (15.6)	< 0.001
	Healthy weight	6190 (54.9)	5769 (55.7)	421 (45.2)	
	Overweight	1603 (14.2)	1460 (14.1)	143 (15.3)	
	Obese	1788 (15.8)	1565 (15.1)	223 (23.9)	

a: most of the times or always

Regarding gender, self –reported asthmatics were significantly more likely to be males as compared to non-asthmatics (65.3% vs. 49.9%, *p* < 0.001). As for nationality, 87.3% of participants were of Saudi Arabian origin and there were significantly more Saudi self-reported than non-Saudi asthmatics (90.7% vs. 87.0%, *p* = 0.001). Fathers of asthmatic adolescents had a lower rate of completing high school (54.6% vs. 59.8%, *p* = 0.003), and families of self-reported asthmatics had lower household incomes in comparison to non-asthmatics (74.8% vs 70.7%, *p* = 0.05). As for comorbidities, only 0.7% had diabetes, with no difference between asthmatics and non-asthmatics. Moreover, self-reported asthmatics had a higher rate of hematological disorders (4.9% vs. 3.6%, *p* = 0.05), allergies-other than asthma (12.2% vs. 4.0%, *p* < 0.001) and genitourinary diseases (2.3% vs 1.1%, *p* = 0.002). Also self-reported asthmatic students had higher levels of BMI and higher rates of obesity (BMI ≥95th percentile) than non-asthmatics (23.9% vs. 15.1%, *p* < 0.001).

### Participants’ dietary patterns and lifestyle factors

Participants’ nutrition and dietary patterns as well as their lifestyle habits, including school behaviors, are presented in Table 2. The majority of students consumed a regular number of main meals and snacks and ordered fast food with no difference between self-reported asthmatics and non-asthmatics. Only 11% consumed more than 5 serving of vegetables or/and fruits per day, while asthmatics intake of soft drinks (63.0% vs 58.7%, *p* = 0.01) and power drinks (26.4% vs 20.9%, *p* < 0.001) was higher and more frequent than non-asthmatics. Also non-asthmatics consumed less milk as compared to self-reported asthmatics (55.3% vs 50.6% did not consume any milk, respectively, *p* = 0.02). As for exercising, no significant difference was found between the two groups, as the majority of the students participated in physical activity less than 3 times a week (71.8%). As for sleeping patterns, self-reported asthmatics more often reported feeling unrefreshed upon awakening in the morning (22.2% vs 24.7%, *p* = 0.09). On the other hand, significant differences in other behaviors were found. Self-reported asthmatics reported smoking more than non-asthmatics (8.0% vs. 6.2%, *p* = 0.04) and were also found to start smoking at an earlier age (for less than 12 years old 8.6% vs. 7.0% and for age between 12 and 18 years 13.7% vs. 9.6%, *p* < 0.001). Also, self-reported asthmatics spent more time on video games (18.1% vs 13.0%, *p* < 0.001) but with no significant differences found in the use of mobile phones. When looking at academic performance, self-reported asthmatics less frequently reported above average academic performance in comparison to non-asthmatics (93.7% vs. 95.8%, *p* = 0.004). Although both groups had similar frequency of school absenteeism (29.6%), there was a significant difference in the reasons for being absent, where asthmatics reported more absenteeism due to sickness or a doctor’s appointment (59.7% vs. 47.4%, *p* < 0.001).

**Table 2:** Difference in diet and nutrition patterns and lifestyle in asthma and healthy controls

		All	Non-asthmatic	Asthmatic	
		(*n* = 11,348) %	(*n* = 10,412) %	(*n* = 936) %	*p*-value
Regular number of main meals per day		9127 (81.3)	8388 (81.4)	739 (79.9)	0.25
Regular number of snacks per day		9489 (84.5)	8711 (84.6)	778 (83.6)	0.42
Fruits and vegetables intake (> 5 servings)		1241 (11.0%)	1123 (10.8)	118 (12.7)	0.08
Frequent^b^ Soft drinks intake		6648 (59.1)	6063 (58.7)	585 (63.0)	0.01
Frequent^b^ Power drinks intake		2400 (21.3)	2155 (20.9)	245 (26.4)	< 0.001
Number of milk drinks per day	0	6136 (54.9)	5668 (55.3)	468 (50.6)	0.02
	1–2	4379 (39.2)	3986 (38.9)	393 (42.5)	
	> 2	662 (5.9)	598 (5.8)	64 (6.9)	
Fast food intake more than twice in the past 7 days		3490 (31.3)	3180 (31.1)	310 (33.6)	0.11
Exercise for 30 minutes in the last 7 days	< 3	8021 (71.8)	7368 (71.9)	653 (70.6)	0.40
	≥3	3154 (28.2)	2882 (28.1)	272 (29.4)	
Sleeping patterns	Often^a^ feels refreshed upon awakening in the morning	2723 (24.5)	2518 (24.7)	205 (22.2)	0.09
Age of onset of cigarette smoking (years)	Never	9233 (82.9)	8512 (83.4)	721 (77.7)	< 0.001
	Less than 12	797 (7.2)	717 (7.0)	80 (8.6)	
	Between 12 and 18	1106 (9.9)	979 (9.6)	127 (13.7)	
Cigarette smoking in the last 30 days		711 (6.4)	637 (6.2)	74 (8.0)	0.04
Video game use > 2 hours daily		1510 (13.4)	1341 (13.0)	169 (18.1)	< 0.001
Mobile phone use > 30 minutes daily		2394 (26.0)	2180 (25.9)	214 (27.8)	0.24
Academic performance during preceding school semester: above average		10,497 (95.6)	9647 (95.8)	850 (93.7)	0.004
Frequent^a^ school absenteeism		3302 (29.6)	3009 (29.4)	293 (31.7)	0.14
Reason for school absenteeism	Was never absent	2292 (20.4)	2156 (20.9)	136 (14.6)	< 0.001
	Sickness or doctor's appointment	5442 (48.4)	4885 (47.4)	557 (59.7)	
	Didn’t want to come	3510 (31.2)	3270 (31.7)	240 (25.7)	

a: most of the times or always

b: daily and more than once

### Relationships with parents and peers and adolescents’ mental health

Participants’ parents and peer relations and mental health are shown in Table 3. The majority perceived their relationship with both parents as being good. On the other hand, students with asthma were more likely to be frequently feeling sad or down for more than 2 weeks in the past 12 months (17.2% vs. 14.1%, *p* = 0.01) and to seek help (12.1% vs. 7.9%, *p* < 0.001). They also tended to be bullied (37.5% vs 30.9%, *p* < 0.001) and to bully (44.5% vs 36.0%, *p* < 0.001) in and outside of the school.

**Table 3:** Psychological State and Parents and Peer Relations in asthma and healthy controls

	All	Non-Asthmatic	Asthmatic	
	(*n* = 11,348) %	(*n* = 10,412) %	(*n* = 936) %	*p*-value
Frequently^a^ felt sad or down for more than 2 weeks (12 months)	1601 (14.4)	1443 (14.1)	158 (17.2)	0.01
Ever sought help for feeling down (12 months)	920 (8.3)	809 (7.9)	111 (12.1)	< 0.001
Having > 1 sibling	1103 (11.8)	997 (11.6)	106 (14.1)	0.04
Perception of relation with father as average	9334 (84.7)	8562 (84.7)	772 (85.2)	0.66
Perception of relation with mother as average	10,329 (93.1)	9487 (93.2)	842 (91.9)	0.15
Frequently feels worried	743 (6.7)	677 (6.6)	66 (7.2)	0.52
Been bullied at least once	3499 (31.5)	3152 (30.9)	347 (37.5)	< 0.001
Was a bully at least once	4081 (36.7)	3671 (36.0)	410 (44.5)	< 0.001

a: most of the times or always

Multivariate analysis showing risk factors for asthma are shown in Table 4. Only variables with significant differences between asthmatics and non-asthmatics were included in the multivariate analysis. It showed that asthmatics compared to non-asthmatics were less likely to be females (OR 0.62; 95% CI, 0.54 to 0.73, *p* < 0.0001). Also father’s level of education was significantly associated with asthma, where those who did not finish high school were more likely to be asthmatics (OR 1.24; 95% CI, 1.08 to 1.42, *p* = 0.002). Asthmatics were more likely to consume power drinks than non-asthmatics (OR 1.18; 95% CI, 1.01 to 1.38, *p* = 0.04). Asthmatics were also found to spend ≥2 h daily on video games more often than non-asthmatics (OR 1.21; 95% CI, 1.01 to 1.46, *p* = 0.04). As for BMI, asthmatics tended to be more overweight or obese (OR 1.33; 95% CI, 1.09 to 1.63, *p* = 0.005 and OR 1.78; 95% CI, 1.50 to 2.13, *p* < 0.0001, respectively). Finally, asthmatics were more likely to be engaged in bullying in the past 12 months, as compared to non-asthmatics (OR 1.20; 95% CI, 1.04 to 1.38, *p* = 0.01).

**Table 4:** Multivariate stepwise logistic regression for factors associated with asthma

	OR (95% CI)	*p*-value
Gender, female	0.62 (0.54–0.73)	< 0.0001
Father’s level of education, did not complete high school	1.24 (1.08–1.42)	0.002
Daily power drink intake, yes	1.18 (1.01–1.38)	0.04
Academic performance during previous school semester, below average	1.24 (0.93–1.67)	0.15
Video games use per day, ≥2 h	1.21 (1.01–1.46)	0.04
Ever bullied (past 12 months), yes	1.20 (1.04–1.38)	0.01
BMI		
<5th percentile	1.17 (0.96–1.43)	0.12
85th - <95th percentile	1.33 (1.09–1.63)	0.005
≥95th percentile	1.78 (1.50–2.13)	< 0.0001

Variables entered into the model (reference categories are underlined): gender (Male, Female), father’s level of education (Did not finish high school, finished high school), BMI (<5th percentile, 5th – <85th percentile, 85th - <95th percentile, ≥95th percentile), smoking during last 30 days (No, Yes), milk intake (cups per day) (0, 1–2, ≥2), daily soft drink intake (No, Yes), daily powder drink intake (No, Yes), fruit and vegetables intake (< 5 times per day, ≥5 times per day), feel fresh when wake up in the morning (No, Yes), video games use per day (< 2 h, ≥2 h), previous school performance (GPA) (Less than good, Good and above), ever bullied in or out of school in the past 12 months (No, Yes), ever been bullied in or out of school in the past 12 months (No, Yes).

## Discussion

Our study described self –reported asthmatic adolescents in KSA and it shed the light on their characteristics, their associated comorbidities, and their health risks. This work is among the largest studies conducted in KSA, and possibly in the region, to characterize adolescent asthmatics with respect to their co-morbidities and lifestyle habits.

Our study revealed that 8.2% of adolescents in KSA are self-reported asthmatics. This is within the range reported in previous studies (8–25%) [21]. Our prevalence showed an increase from the previous nationwide study in 2013 conducted by Moradi-Lakeh et al. who reported the prevalence to be 4.02% [26]. Although there were no comprehensive programs for early diagnosis and appropriate management of asthma for children and adults, the impact of several programs such as the Asthma Insights and Reality in the Kingdom of Saudi Arabia (AIRKSA) launched in 2008 by the Ministry of Health may have had a role in increasing asthma awareness and contributing to the current increase in diagnosis [27]. Other possible explanations could be related to smoking. Asthma has been linked to smoking, and the prevalence of smoking among Saudi citizens is on the rise [28, 29]. Recent epidemiological studies in Saudi Arabia revealed an increasing prevalence of asthma the past three decades that may be attributed to rapid lifestyle changes related to the modernization of Saudi society, changes in dietary habits, and exposure to environmental factors, such as indoor allergens, dust, sand storms and tobacco. Additionally, awareness of the disease can affect its prevalence and severity in a population [30].

Similar to other studies [4, 31, 32], our study revealed gender differences, with male adolescents being more likely to be self-reported asthmatics than females. This male preponderance to asthma has been explained by differential growth of lung/airway size, and immunological differences [33].

Similar to others’ reports [15, 34, 35], we found that self –reported asthma was associated with lower socioeconomic backgrounds. This is very important to consider as higher-educated parents may have more asthma knowledge that enables them to provide better care for their asthmatic children and be better equipped to provide the appropriate care [36].

The co-morbidities and various health conditions associated with asthma can significantly affect asthma and be affected by asthma [13, 37]. Our results revealed that self –reported asthma is more likely to be found in children and adolescents who are overweight or obese. The prevalence of asthma has increased worldwide among obese children, adolescents and adults regardless of ethnic origins [38–40]. In a study conducted in KSA in 2014, there was an association between asthma and obesity in Saudi children [41]. This relationship between obesity and asthma has been discussed in several studies, and is currently considered to be a risk factor for asthma [42]. Overweight and obese subjects tend to develop asthma symptoms more due to their shallower breathing patterns as compared to healthy weight subjects. This could be due to more than one aspect; overweight subjects have more fat tissue keeping less space for their lungs to inflate, and they have more leptin and other hormones released from fat tissue resulting in higher inflammation in the airways [43]. Similarly obese subjects were found to have the asthma-like phenotype [42].

Other risk factors instituted for having asthma and allergic disorders was diet [44]. As the environmental changes, eating pattern changes, and lifestyles alterations have sewed up, asthma prevalence was concomitantly on increase [45]. Notably, the traditional diet constituting of high fiber diet and high milk intake have been transformed to a modern diet rich in preserved refined sugar and containing less dietary fiber [44].

Self-reported asthmatics’ intake of power drinks is significantly higher than their healthy counterparts. There is scarcity of information in the literature to address this habit, and the relationship between asthmatics and consumption of power drinks remains vague. Nevertheless, many studies suggest that asthmatic children and adolescents are more likely to fall asleep during the day and to feel tired and powerless due to the wheezing that occurs at night and their inability to sleep well [46, 47]. We speculate whether this tiredness and sleepiness and lack of power felt by asthmatic patients are the driving factors for the consumption of more power drinks and the need for increased caffeine intake. Interestingly, energy drinks are known to be high in caffeine and sugar, in addition to other pharmaceutical and herbal stimulants [48], and this may raise dramatically blood glucose leading to unhealthy food craving, fatigue and irritation [49]. Thus asthmatics may enter an unhealthy vicious cycle of consuming more sugar that will increase their BMI leading to more power drinks consumption to recover their energy. The consequent result of increase in body weight will exacerbate their asthma symptoms and disturb further their sleep. In addition, power drinks are known to be rich in caffeine, a central nervous system stimulant from the xanthine class known to improve airway function and relieve asthma symptoms and exacerbation [50]. We wonder if this physiologic effect has in any way played an indirect role in the high consumption of power drinks among our asthmatics.

As for consumption of milk, the relation between asthma and drinking milk did not show on the multivariate analysis. This is not in agreement with the report of Hijazi et al. in 2000 [51], which showed an inverse linear relationship between asthma and the intake of milk in Saudi children. While studies have shown a protective effect of farm milk consumption on asthma [52, 53], based on which clinicians may be advising their asthmatic patients to drink more milk, our study failed to show any effect or correlation between drinking milk and asthma disease.

The literature shows strong links between asthma and lifestyle factors, school behaviours, children’s’ psychological state and violence. Poongadan et al. showed an increasing association between sedentary lifestyle and asthma, and reported that asthmatics watch more TV and have increasing mental stress [54] .This was not the case in our study and this association was not significant.

Although self –reported asthma has been found to be more significantly associated with depressive disorders, anxiety and being bullied [55, 56], our self-reported asthmatics were not found to be more psychologically affected nor were they more violent or bullied in the multivariate analysis. We speculate whether this could be due to a peer effect that is more supportive than bullying. This is supported by the development of prevention programs in SA that focused on education and increasing the awareness of bullying and its negative impacts [57, 58].

This study is an eye opener to physicians who care for and provide management to adolescents with asthma and is a reminder of the importance of viewing a health condition and an individual holistically. It is prudent to bear in mind the individual’s health correlates and lifestyle as this can impact the disease and affect the quality of life. Assessment and management of asthma calls for a comprehensive care that conveys both medical treatment and healthy habits and lifestyle.

### Limitations

Our study had some limitations that ought to be addressed; asthma was identified by adolescents’ self-report. Some adolescents, especially the younger ones, may not have necessarily been familiar with having a diagnosis of asthma. Therefore, an element of underreporting may exist. Furthermore, due to the nature of this study, we have been able to identify correlates; causality may not be inferred.

## Conclusion

Asthma is an increasingly prevalent disease in Saudi Arabia, affecting males more than females and adolescents of lower socioeconomic backgrounds. Being a chronic disease, there is a need to address asthma awareness specifically in relation to adolescents’ eating and drinking habits. More education to asthmatics and their families is needed to address the risks or protective effects of some dietary items such as power drinks and milk.

## Data Availability

Original database used in the original study (Jeeluna Study).

## Abbreviations

KSA: Kingdom of Saudi Arabia
MOE: Ministry of Education
BMI: Body Mass Index
OR: Odds Ratio
CI: Confidence Interval
AIRKSA: Asthma Insights and Reality in the Kingdom of Saudi Arabia
